# Comparison of an AI Chatbot With a Nurse Hotline in Reducing Anxiety and Depression Levels in the General Population: Pilot Randomized Controlled Trial

**DOI:** 10.2196/65785

**Published:** 2025-03-06

**Authors:** Chen Chen, Kok Tai Lam, Ka Man Yip, Hung Kwan So, Terry Yat Sang Lum, Ian Chi Kei Wong, Jason C Yam, Celine Sze Ling Chui, Patrick Ip

**Affiliations:** 1 Department of Paediatrics and Adolescent Medicine, School of Clinical Medicine, Li Ka Shing Faculty of Medicine, University of Hong Kong, Room 115, 1/F, New Clinical Building, Queen Mary Hospital, 102 Pokfulam Road, Hong Kong SAR, China (Hong Kong), 852 22554090, 852 28551523; 2 Department of Social Work Administration, University of Hong Kong, Hong Kong SAR, China (Hong Kong); 3 Department of Pharmacology and Pharmacy, Li Ka Shing Faculty of Medicine, University of Hong Kong, Hong Kong SAR, China (Hong Kong); 4 School of Pharmacy, Medical Sciences Division, Macau University of Science and Technology, Macau, China; 5 School of Pharmacy, Aston University, Birmingham, United Kingdom; 6 Department of Ophthalmology and Visual Sciences, Chinese University of Hong Kong, Hong Kong SAR, China (Hong Kong); 7 School of Nursing, University of Hong Kong, Hong Kong SAR, China (Hong Kong)

**Keywords:** AI chatbot, anxiety, depression, effectiveness, artificial intelligence

## Abstract

**Background:**

Artificial intelligence (AI) chatbots have been customized to deliver on-demand support for people with mental health problems. However, the effectiveness of AI chatbots in tackling mental health problems among the general public in Hong Kong remains unclear.

**Objective:**

This study aimed to develop a local AI chatbot and compare the effectiveness of the AI chatbot with a conventional nurse hotline in reducing the level of anxiety and depression among individuals in Hong Kong.

**Methods:**

This study was a pilot randomized controlled trial conducted from October 2022 to March 2023, involving 124 participants allocated randomly (1:1 ratio) into the AI chatbot and nurse hotline groups. Among these, 62 participants in the AI chatbot group and 41 in the nurse hotline group completed both the pre- and postquestionnaires, including the GAD-7 (Generalized Anxiety Disorder Scale-7), PHQ-9 (Patient Health Questionnaire-9), and satisfaction questionnaire. Comparisons were conducted using independent and paired sample *t* tests (2-tailed) and the *χ*^2^ test to analyze changes in anxiety and depression levels.

**Results:**

Compared to the mean baseline score of 5.13 (SD 4.623), the mean postdepression score in the chatbot group was 3.68 (SD 4.397), which was significantly lower (*P*=.008). Similarly, a reduced anxiety score was also observed after the chatbot test (pre vs post: mean 4.74, SD 4.742 vs mean 3.4, SD 3.748; *P*=.005), respectively. No significant differences were found in the pre-post scores for either depression (*P*=.38) or anxiety (*P*=.19). No statistically significant difference was observed in service satisfaction between the two platforms (*P*=.32).

**Conclusions:**

The AI chatbot was comparable to the traditional nurse hotline in alleviating participants’ anxiety and depression after responding to inquiries. Moreover, the AI chatbot has shown potential in alleviating short-term anxiety and depression compared to the nurse hotline. While the AI chatbot presents a promising solution for offering accessible strategies to the public, more extensive randomized controlled studies are necessary to further validate its effectiveness.

## Introduction

An estimated 1 in 7 people in Hong Kong experience a common mental disorder in their lifetime, with anxiety and depression being the two most common mental health issues in Hong Kong, accounting for 19% and 14% among the generation population, respectively [1,2]. Nearly three-quarters of individuals experiencing a mental health disorder do not seek professional help for various reasons such as high cost of services, fear of judgment, or stigma [3]. To address these potential problems, artificial intelligence (AI) chatbots have been incorporated into digital interventions, particularly web- and smartphone-based apps, to enhance user experience and optimize individualized mental health [4,5]. Chatbots can provide a safe and confidential space for people to seek help for their mental health concerns. Additionally, they are available at any time throughout a study, making them a convenient option for those who may lack access to traditional mental health services or require support beyond regular business hours. Further efforts toward strengthening implementation of chatbots are needed, and their application to the general public should be explored to improve perceptions of general mental health and increase awareness of the importance of premedical interventions in the future [6].

Chatbot models have been developed in various countries and are widely used in psychology and clinical practice. For example, Tess, a psychological AI service, has been tailored to provide immediate assistance to caregiving specialists, individuals receiving care, and family caregivers within a nonprofit organization in the United States and Canada [7]. Among university students, chatbot applications have showed a significant decrease in anxiety symptoms [8]. Anna, an AI-driven chatbot, simulates the function of a therapist and offers gamified versions of evidence-based activities drawn from various therapeutic approaches. Participants who engaged in activities facilitated by Anna provided more detailed responses containing a higher number of positive relational terms [9]. A review of users’ opinions, satisfaction, and attitudes regarding depression and anxiety chatbot apps found that users felt supported and confident while they used apps that were easy to navigate, affordable, and free of cost [10]. In Hong Kong, the first AI-driven Cantonese psychological support tool, the Pai.ACT mobile app, was developed for parents of children with special education needs [11]. However, there is currently a lack of evidence on the effectiveness of AI chatbots in tackling mental health problems in the general public.

Given the risk of anxiety and depression in Hong Kong and the limited availability of mental health services during the epidemic, this study aimed to compare the effectiveness of the developed AI chatbot with that of a conventional nurse hotline in alleviating psychological anxiety and depression in the general public. Additionally, this study also aimed to understand user satisfaction with chatbot usage and gather preliminary data for larger randomized controlled trials to advocate for the AI chatbot as a psychological support tool for individuals seeking information on the chatbot platform.

## Methods

### Recruitment

This study was designed as a randomized controlled trial (RCT) with two parallel groups. Participants were parents recruited through two school principal’s networks. The inclusion criteria included (1) being able to use smartphones proficiently to interact with the AI chatbot, (2) being fluent in Chinese language used in the study to effectively communicate with the AI chatbot, and (3) providing an electronic consent form. Parents who were unwilling or unable to commit to the entire research process and had difficulties in reading and understanding Chinese were excluded. Invitation letters were sent to kindergarten and primary school principal groups from chief schools, and 124 parents responded to this study and provided an electronic consent form. Participants were then randomly allocated in a 1:1 ratio to either the AI chatbot or the nurse hotline group using block randomization with blocks of four. Participants immediately filled out the posttest questionnaires after communicating with the AI chatbot or nurse. Those who did not respond immediately received reminders until they completed the posttest questionnaires. Participants with risky mental health problems were followed up by phone calls and were encouraged to ask to connect with necessary medical services and mental health clinical departments.

### Ethical Considerations

This study was approved by the Institutional Review Board of the University of Hong Kong/Hospital Authority Hong Kong West (approval number: UW21-344). Informed consent was obtained from all participants in the study, ensuring that they were fully aware of the nature and possible consequences of their participation. Participants were informed that they had the right to opt out at any time without penalty. The original consent and approval covers secondary analysis without additional consent. Data collected from participants were deindentified to ensure privacy and confidentiality. Identifying information, including names, initials, or hospital numbers, was removed from all datasets to protect participant identities. Participation was voluntary, and individuals were informed that no financial or material incentives would be provided. Consolidated Standards of Reporting Trials (CONSORT) reporting guideline was adhered to in this study [12].

### AI Chatbot Development Procedures

The development of the AI chatbot involved 7 key steps. First, the developer analyzed requirements by understanding common queries and focusing on providing accurate information to the public and assisting nurses. Relevant data was then collected from credible sources, and natural language processing (NLP) algorithms were used to train a robust model on diverse questions and answers. By integrating large language models, the chatbot could provide detailed responses. Next, the developer designed and developed the backend infrastructure, including databases, application programming interfaces, and integration with University of Hong Kong’s website and WhatsApp. Extensive testing and validation ensured the chatbot’s accuracy and user-friendliness, with a beta version tested by selected users. Finally, the chatbot was deployed on University of Hong Kong’s website and made accessible through WhatsApp, with a nurse hotline version developed to assist health care professionals in handling public inquiries.

The AI chatbot system comprises several essential components to provide accurate and relevant information. It begins with context identification, where the chatbot analyzes keywords and phrases to understand the user’s query, ensuring accurate interpretation and relevant response. Next, the system conducts query analysis using NLP techniques to determine the user’s intent and extract specific details such as dates, locations, and vaccination information. The query response system then leverages the trained NLP model and large language model–generated responses to provide accurate and contextually appropriate answers tailored to users’ needs. Finally, the chatbot relies on a regularly updated and comprehensive dataset containing the latest information on COVID-19 vaccinations, government regulations, and health care procedures, ensuring the delivery of reliable and accurate responses.

### Questionnaires

The Patient Health Questionnaire-9 (PHQ-9) is a self-assessment tool consisting of 9 questions that measure the frequency and severity of depressive symptoms over the last 2 weeks. Each question is derived from the *Diagnostic and Statistical Manual of Mental Disorders, Fourth Edition* (*DSM-IV*) criteria and is rated on a scale of 0 (not at all) to 3 (almost daily) [13]. The Generalized Anxiety Disorder Scale-7 (GAD-7) is a 7-item self-report scale that evaluates the frequency and severity of anxious thoughts and behaviors during the last 2 weeks. Items are based on the diagnostic criteria of the *DSM-IV* and scored from 0 (not at all) to 3 (nearly every day) [14]. The service satisfaction survey is a 3-item questionnaire for participants to report their degree of satisfaction after using the two platforms. The rating score ranges from 0 to 10, representing an increasing satisfaction from not at all likely to extremely likely. Based on the standardized cutoff that determines the severity of anxiety and depression, participants were divided into two groups: the no-risk group (total score<4) and the risk group (total score>4) [15].

### Statistical Analysis

Quantitative variables were tested for normal distribution using the Kolmogorov-Smirnov test. Normally distributed continuous variables were expressed as mean (SD) and categorical variables were expressed as numbers and percentages. A per-protocol analysis was performed for all outcomes when comparing the 2 groups. The independent *t* test (2-tailed) was employed to compare the preanxiety or predepression, postanxiety or postdepression, pre-post difference scores, and service satisfaction between the AI chatbot and nurse hotline groups, respectively. The paired *t* test (2-tailed) was used to compare scores before and after group chat. Linear regression was conducted to analyze the difference in posttest scores between the AI chatbot and nurse hotline groups adjusted by pretest scores. Categorical variables were compared with the *χ*^2^ test. All analyses were conducted using SPSS software (version 29.0; IBM Corp).

## Results

**Figure 1.**
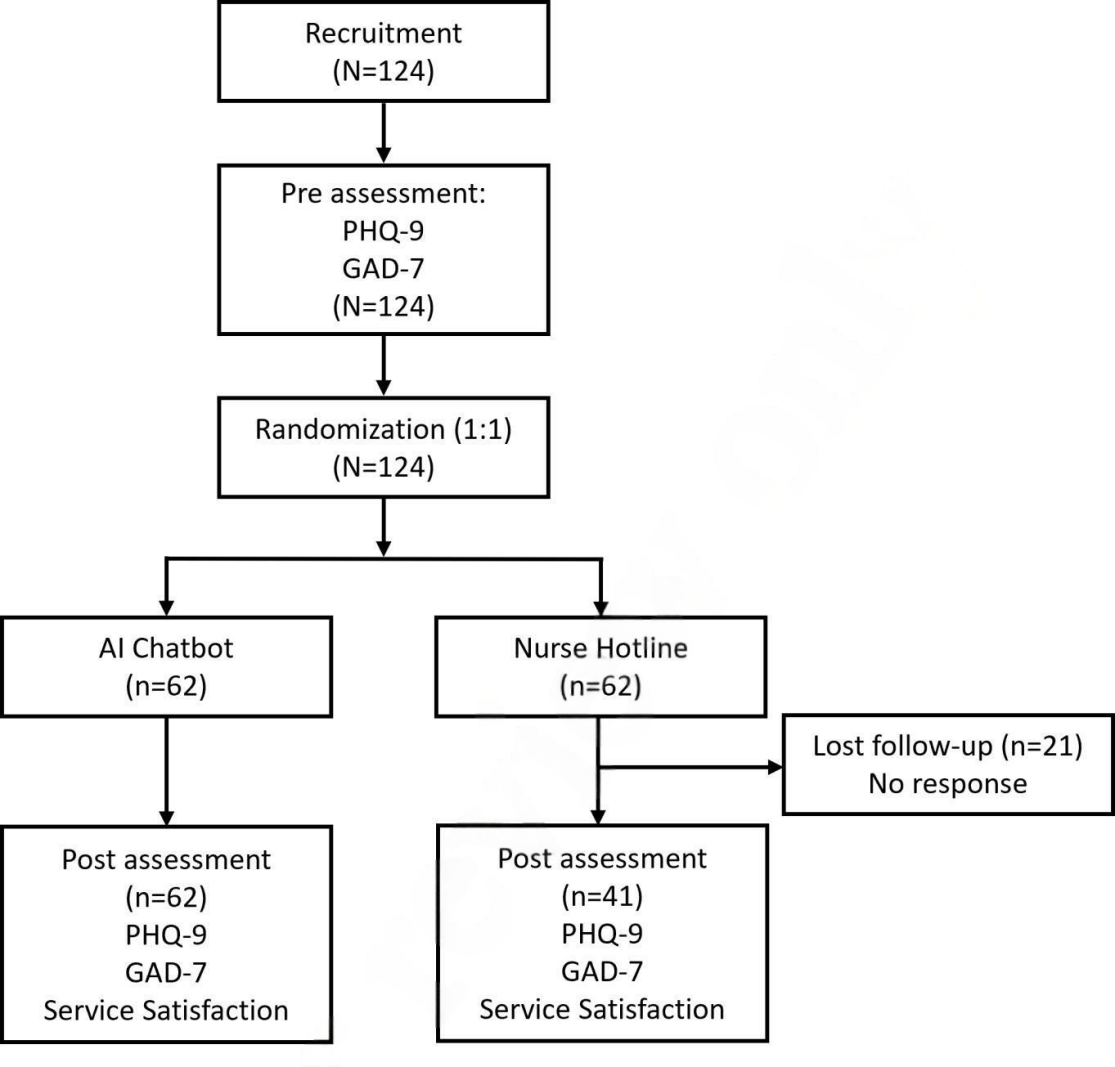
Diagram of the study design. AI: artificial intelligence. PHQ-9: Patient Health Questionnaire-9; GAD-7: Generalized Anxiety Disorder Scale-7.

**Figure 2.**
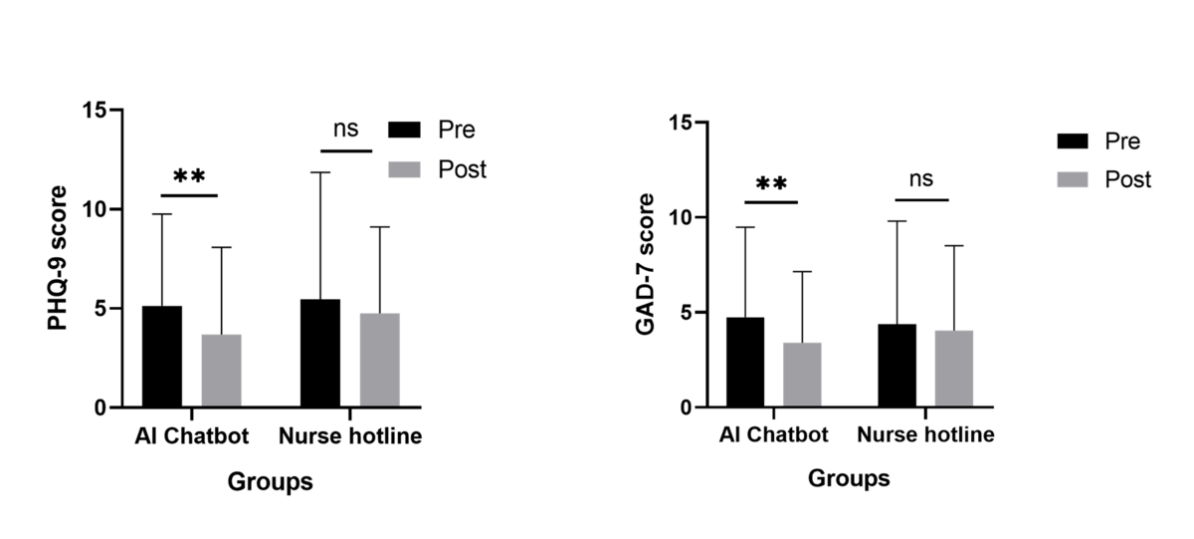
Comparison of anxiety and depression scores within groups pre- and postintervention. GAD-7: Generalized Anxiety Disorder Scale-7; PHQ-9: Patient Health Questionnaire-9; ns: nonsignificant. **P<.05.

From October 2022 to March 2023, all the participants (N=124) answered prequestionnaires, and 62 (62/62, 100%) participants in the AI chatbot group and 41 (41/62, 66.1%) in the nurse hotline group completed both the pre- and postquestionnaires (Figure 1). Comparisons within groups before and after the test are displayed in Table 1 and Figure 2. Compared to the mean baseline score of 5.13 (SD 4.623), the mean postdepression score 3.68 (SD 4.397) was significantly lower in the chatbot group (*P*=.008). Similarly, a reduction in anxiety score was also observed after using the chatbot test (pre vs post: mean 4.74, SD 4.742 vs mean 3.4, SD 3.748; *P*=.005).

**Table 1.** Comparison of the anxiety and depression scores within groups before and after the test.

Questionnaires and groups (N=124)^a^	Pretest score, mean (SD)	Posttest score, mean (SD)	*P* value
PHQ-9^b^			
AI^c^ chatbot (n=62)	5.13 (4.632)	3.68 (4.397)	.008
Nurse hotline (n=41)	5.46 (6.4)	4.76 (4.346)	.28
GAD-7^d^			
AI chatbot (n=62)	4.74 (4.742)	3.4 (3.748)	.005
Nurse hotline (n=41)	4.37 (5.439)	4.05 (4.455)	.63

a n=21 participants were lost to follow up.

b PHQ-9: Patient Health Questionnaire-9.

c AI: artificial intelligence.

d GAD-7: Generalized Anxiety Disorder Scale-7.

Table 2 shows there were no statistically significant differences at baseline between groups in depression (*P*=.76) and anxiety scores (*P*=.71). Although the postdepression and postanxiety scores in the chatbot group were slightly lower than those in the nurse hotline group, there was no statistical difference (all *Ps*>.05). No statistically significant difference was observed in service satisfaction between the 2 service platforms (*P*=.32). In the nurse hotline group, there were no notable differences in depression and anxiety scores before and after the test.

**Table 2.** Comparison of the before and after PHQ-9^a^, GAD-7^b^, and service satisfaction scores between the AI chatbot and nurse hotline groups.

Variables	AI chatbot scores (n=62), mean (SD)	Nurse hotline scores (n=41), mean (SD)	*P* value^c^
Pre–PHQ-9	5.13 (4.63)	5.46 (6.4)	.76
Post–PHQ-9	3.68 (4.40)	4.76 (4.35)	.43
Pre–GAD-7	4.74 (4.74)	4.37 (5.44)	.71
Post–GAD-7	3.40 (3.75)	4.05 (4.46)	.22
Service satisfaction	16.11 (6.88)	17.39 (5.59)	.32

a PHQ-9: Patient Health Questionnaire-9.

b GAD-7: Generalized Anxiety Disorder Scale-7.

c Independent *t* test.

No significant variations were observed in the pre-post score differences for either depression (*P*=.38) or anxiety scores (*P*=.19) between the 2 groups (Table 3).

**Table 3.** Comparison of the pre-post anxiety and depression score differences between the artificial intelligence (AI) chatbot and nurse hotline groups.

Variables	Groups		
	AI chatbot (n=62), mean (SD)	Nurse hotline (n=41), mean (SD)	*P* value
Pre-post differences			
PHQ-9^a^	−1.452 (4.203)	−0.707 (4.161)	.38
GAD-7^b^	−1.339 (3.635)	−0.317 (4.15)	.19

a PHQ-9: Patient Health Questionnaire-9.

b GAD-7: Generalized Anxiety Disorder Scale-7.

c Independent *t*-test was used to calculate *P* value.

The various test groups were not associated with the postdepression score (*β*=−.902, *P*=.18) or the postanxiety score (*β*=−.845, *P*=.17), after adjusting the prescores (Table 4).

**Table 4.** Regression analysis on the posttest score between the artificial intelligence chatbot and nurse hotline adjusted by pretest score (n=103).

Questionnaires and groups	β estimate	Standard error	*t* test (*df=100)*	*P* value
Post–PHQ-9^a^ (ref: nurse group)				
Pre–PHQ-9	0.528	0.0615	8.591	<.001
Chatbot	−0.902	0.6720	−1.342	.18
Post–GAD-7^b^ (ref: nurse group)				
Pre–GAD-7	0.531	0.0604	8.787	.001
Chatbot	−0.845	0.6148	−1.375	.17

a PHQ-9: Patient Health Questionnaire-9.

b GAD-7: Generalized Anxiety Disorder Scale-7.

Based on the PHQ-9 and GAD-7 cutoff, the pre- and postscores were categorized into no-risk group and risk groups. No significant differences were observed in the prevalence of anxiety and depression before and after using the nurse hotline or AI chatbot (Table 5).

**Table 5.** Comparison of the anxiety and depression prevalence before and after using the nurse hotline and artificial intelligence (AI) chatbot among participants.

Groups	No-risk group (score≤4), n (%)	Risk group (score>4), n (%)	*P* value
Pre–PHQ-9^a^			
Nurse hotline (n=41)	27 (65.9)	14 (34.1)	.20
AI chatbot (n=62)	33 (53.2)	29 (46.8)	
Post–PHQ-9^a^			
Nurse hotline (n=41)	26 (63.4)	15 (36.6)	.33
AI chatbot (n=62)	45 (72.6)	17 (27.4)	
Pre–GAD-7^b^			
Nurse hotline	27 (65.9)	14 (34.1)	.34
AI chatbot	35 (56.5)	27 (43.5)	
Post–GAD-7^b^			
Nurse hotline	27 (65.9)	14 (34.1)	.47
AI chatbot	45 (72.6)	17 (27.4)	

a PHQ-9: Patient Health Questionnaire-9.

b GAD-7: Generalized Anxiety Disorder Scale-7.

## Discussion

### Principal Results

This study is among the first in Hong Kong to compare the effectiveness of AI chatbots and nursing hotlines in addressing mental health–related inquiries. The AI chatbot proved effective in reducing anxiety and depression during the COVID-19 outbreak and was comparable to the conventional nursing hotline. It suggests the potential use of AI chatbots as a complementary approach in mental health interventions, particularly when AI chatbots can provide timelier and uninterrupted 24-hour support to users in need during the challenging periods such as a pandemic.

Previous research comparing the effectiveness of the AI chatbot and humans in improving mental health is controversial. Our study found that both the AI chatbot and nurse hotline had similar effects in alleviating depression and anxiety. The results are consistent with another study comparing the text-only chatbot and voice-based digital human interactions as responders to mental health questions, which reported that the text-only chatbot was more user-friendly, although there were no significant differences in electroencephalography measurements [16]. No differences were observed in another RCT comparing the effects of AI chatbot (ie, ChatGPT-3.5) and nurse education on preoperative anxiety reduction among groups [17]. However, one study comparing physician and AI chatbot responses to patient questions posted on a public social media forum found that the chatbot generated higher quality and empathetic responses to patient questions than physicians [18]. Additionally, the follow up process took less time for the nurse hotline group compared to the chatbot group. This was primarily because participants in the nurse hotline group were quicker in responding to follow up questionnaires while the chatbot group typically required more than one attempt to gather responses. It is feasible to use the AI chatbots to provide instant information to users about mental health knowledge and basic strategies to dissipate their temporary anxiety and depressed mood. However, the AI chatbot can only be a facilitator, and humans will not be replaced in the long-term professional treatment. The sample size in the above-mentioned studies is very small, which may have limited the power of statistical analysis. Therefore, more RCTs with larger sample sizes are necessary to confirm differences between the AI chatbot and human-based services.

Our study showed a significant reduction in the levels of anxiety and depression after using the AI chatbot. This finding is supported by a study from Argentina, which showed a substantial decrease in anxiety symptoms in university students using *Tess* [8]. Another study reported that the use of the Elomia chatbot for 4 weeks resulted in a significant decrease in the symptoms of anxiety and depression along with reduced negative effects [19]. AI chatbots can offer information to users in a quick and easy format. They can also be programmed to answer specific questions about a certain condition, such as what to do during a medical crisis or what to expect during a medical procedure. The AI chatbot could lower the barriers to care by helping patients access help more quickly and efficiently [20]. AI chatbots appear to have a positive impact on depression and anxiety in a wide range of populations, including university students [8], short-course treatment patients [21], and preoperative patients [17]. The potential benefit of chatbot–assisted mental health support is that it can provide helpful information about depressive moods, especially for users who have difficulty in communicating emotions to other humans [22]. Besides, the chatbot offers real-time relief, emotional support, and instant messages on basic knowledge about health-related questions. AI chatbots can help reduce anxiety and depression by providing accessible, anonymous, consistent, and immediate emotional support. They serve as a valuable supplement to conventional therapy and mental health care, offering users a safe space to express their feelings and find effective coping strategies.

Our study differs from previous ones by addressing a research gap in the broader population. Earlier studies primarily concentrated on specific groups, such as college students aged 18-33 [8], younger individuals aged 19-23 [19], and older adults aged 60 years [17]. The sample size in our study (N=124) aligns with past studies, which typically included approximately 100 participants. A study with a larger sample size of 412 younger participants [19] also found AI chatbots to be effective in alleviating anxiety and depression symptoms. Notably, our study was conducted during the COVID-19 pandemic, unlike previous studies, which were conducted under normal circumstances. Hence, additional research is necessary to understand the efficacy and impact of AI chatbots across different age groups, demographics, and environmental contexts.

Our study was carried out during the COVID-19 outbreak, revealing that AI chatbot interventions are versatile and can be applied in various situations, such as during future pandemics or for disseminating information to prevent other diseases. There is great potential in AI chatbot designing and application during an epidemic like COVID-19 [23]; AI chatbots not only help in reducing depression and anxiety at any given time but also can be embedded into the health care systems, to help patients describe their symptoms and provide preliminary guidance on whether they need to seek medical attention [24]. This can assist in triaging patients based on the severity of their symptoms. They can help patients book appointments with health care providers, reducing the burden on administrative staff. AI Chatbots can send reminders to patients to take their medications on time, improving medication adherence [24]. They can provide ongoing emotional support and coping strategies for individuals dealing with mental health issues like anxiety, depression, and stress. This can complement traditional therapy and make mental health care more accessible. Chatbots can also deliver personalized health education to patients, helping them understand their conditions, treatment options, and preventive measures.

The findings of this study comparing AI chatbots and nurse hotlines in alleviating the levels of anxiety and depression during the COVID-19 pandemic can be generalized to a certain extent, given that the general population was the target group. This broad target population ensures that the results are applicable to a wide range of individuals who may have experienced similar mental health challenges during the pandemic. However, the generalizability may be limited by factors such as the specific characteristics of the sample used in the study, including their demographics and the severity of their anxiety and depression symptoms. Additionally, the dynamic nature of AI chatbots, which can be influenced by various factors such as the phrasing of questions and ongoing optimization processes, may also affect the generalizability of the results. The study’s design and methodology provide a solid foundation for replicability. The use of an RCT framework, which is a robust design for evaluating interventions, increases the likelihood that similar studies can be conducted with comparable results. The clear documentation of the study’s procedures, including the recruitment process, data collection methods, and statistical analysis techniques facilitates replication by other researchers. Furthermore, the study’s focus on a common and significant issue—mental health during a pandemic—ensures that there is a continued interest and relevance in conducting similar studies. However, replicability may be challenged by the need for access to similar populations and resources, as well as the evolving nature of AI chatbot technology, which may require adaptation of the intervention for future studies.

Although AI chatbots have a potential complementary function in mental health and health-related fields, certain drawbacks must be considered. For example, the safety and privacy concerns remain unclear [25]. Chatbots are unable to deliver the emotional support and personal bond that a certified mental health expert may provide. They cannot deliver a diagnosis or management strategy for mental health disorders. Therefore, it is crucial to use AI chatbots as a complement to, rather than a substitute for, specialized mental health services. Moreover, further refinement to the AI chatbot is necessary for establishing an integrated system for recognition-alert-reporting system for mental health problems. After early detection and identification of depression and anxiety, the AI chatbot should be designed to automatically send self-regulation advice to the users to handle their negative emotions as well as send reminder messages to encourage them to seek medical services. Additionally, the question coverage of the AI chatbot is limited; it covers common mental health–related questions that may overlook the scope of other health problems. The scope of the population using chatbots should be expanded among the public. Further studies are required to draw solid conclusions about the effectiveness and safety of chatbots.

### Limitations

One of the limitations of our study is the small sample size. Toward the end of the COVID-19 outbreak, there was a notable lack of enthusiasm among parents to participate in this study. Only two school principal groups were involved, which may have introduced a selection bias. Additionally, the study period was too short to recruit sufficient participants; however, the preliminary results showed a significant reduction in anxiety and depression after using the AI chatbot, supporting the applicability of using AI chatbots to relieve negative emotions during the next epidemic crisis. Demographic data and other confounding factors were not collected due to privacy concerns; these factors will be included in the following larger randomized control study.

### Conclusions

The AI chatbot is as effective as the nurse hotline in answering mental health–related questions. Besides, the AI chatbot can reduce short-term anxiety and depression levels compared to the nurse hotline. The developed AI chatbot shows promise as a complementary tool to provide available intervention strategies to the public, particularly for families with limited access to mental health services. This study provides insights into the potential application of using AI chatbots to provide immediate information to the public and mitigate public emotional distress during future epidemic emergencies.

## Supplementary material

10.2196/65785 Checklist 1 CONSORT-eHEALTH checklist (V1.6.1).

## Abbreviations

AI: artificial intelligence
CONSORT: Consolidated Standards of Reporting Trials
*DSM-IV*: *Diagnostic and Statistical Manual of Mental Disorders, Fourth Edition*
GAD-7: Generalized Anxiety Disorder Scale-7
NLP: natural language processing
PHQ-9: Patient Health Questionnaire-9
RCT: randomized controlled trial
