# Supplementary material for: Comparison of an AI Chatbot With a Nurse Hotline in Reducing Anxiety and Depression Levels in the General Population: Pilot Randomized Controlled Trial
Source: JMIR Hum Factors. 2025 Mar 6;12:e65785. doi: 10.2196/65785 (PMC11906115; doi:10.2196/65785)
Supplement: Checklist 1 [file humanfactors-v12-e65785-s001.pdf]

CONSORT-EHEALTH Checklist V1.6.2 Report	Manuscript Number	65785
**(based on CONSORT-EHEALTH V1.6), available at [http://tinyurl.com/consort-ehealth-v1-6].**		
**Date completed**		
1/13/2025 23:38:19		
**by**		
Patrick Ip		
AI Chatbot Versus Nurse Hotline for the Level of Anxiety and Depression in General Population: a Pilot Study		
TITLE		
**1a-i) Identify the mode of delivery in the title**		
"like this: the AI chatbot"		
**1a-ii) Non-web-based components or important co-interventions in title**		
**1a-iii) Primary condition or target group in the title**		
"like this: AI Chatbot Versus Nurse Hotline for the Level of Anxiety and Depression in General Population: a Pilot Study"		
ABSTRACT		
**1b-i) Key features/functionalities/components of the intervention and comparator in the METHODS section of the ABSTRACT**		
"This was a pilot randomized controlled trial conducted from Oct 2022 to March 2023, involving 124 participants allocated randomly (1:1 ratio) into the AI chatbot group and nurse hotline group. 62 participants in the AI chatbot group and 41 in the nurse hotline group completed both the pre-and post-questionnaires"		
**1b-ii) Level of human involvement in the METHODS section of the ABSTRACT**		
**1b-iii) Open vs. closed, web-based (self-assessment) vs. face-to-face assessments in the METHODS section of the ABSTRACT**		
**1b-iv) RESULTS section in abstract must contain use data**		
**1b-v) CONCLUSIONS/DISCUSSION in abstract for negative trials**		
INTRODUCTION		
**2a-i) Problem and the type of system/solution**		
"like this: In light of the risk of anxiety and depression in Hong Kong and the limited mental health services during epidemic, this study aimed to compare the effectiveness of the developed AI chatbot and the conventional nurse hotline in alleviating psychological anxiety and depression in the general public. Another aim was to understand user satisfaction with chatbot usage as well as to gather initial data for larger randomized controlled trials to advocate for the AI chatbot as a psychological support tool for individuals seeking information on the chatbot platform. "		
**2a-ii) Scientific background, rationale: What is known about the (type of) system**		
The introduction part: paragraph 1 & 2		
**Does your paper address CONSORT subitem 2b?**		
"like this: this study aimed to compare the effectiveness of the developed AI chatbot and the conventional nurse hotline in alleviating psychological anxiety and depression in the general public. Another aim was to understand user satisfaction with chatbot usage as well as to gather initial data for larger randomized controlled trials to advocate for the AI chatbot as a psychological support tool for individuals seeking information on the chatbot platform. "		
METHODS		
**3a) CONSORT: Description of trial design (such as parallel, factorial) including allocation ratio**		
"like this: 124 parents responded to this study and provided an electronic consent form. Participants were then randomly allocated with a 1:1 ratio into either the AI chatbot or the nurse hotline group with a block randomization of four"		
**3b) CONSORT: Important changes to methods after trial commencement (such as eligibility criteria), with reasons**		
Not applicable, no important changes to methods after trial commencement		
**3b-i) Bug fixes, Downtimes, Content Changes**		
**4a) CONSORT: Eligibility criteria for participants**		
"like this: The inclusion criteria include (1) being able to use smartphones proficiently to interact with the AI chatbot, (2) being fluent in the Chinese used in the study to effectively communicate with the AI chatbot, and (3) providing an electronic consent form. Parents who were unwilling or unable to commit to the entire research process and had difficulties in reading and understanding Chinese were excluded. "		
**4a-i) Computer / Internet literacy**		
**4a-ii) Open vs. closed, web-based vs. face-to-face assessments:**		
"like this: From Oct 2022 to March 2023, all the participants (N=124) answered pre-questionnaires, and 62 participants in the AI chatbot group and 41 in the nurse hotline group completed both the pre-and post-questionnaires (Figure 1). Participants immediately filled out the post-test questionnaires after communicating with the AI chatbot or nurse."		
**4a-iii) Information giving during recruitment**		
**4b) CONSORT: Settings and locations where the data were collected**		
"like this: Participants were parents recruited through two school principal's networks. Invitation letters were sent to kindergarten and primary school principal groups from chief schools, and 124 parents responded to this study and provided an electronic consent form."		
**4b-i) Report if outcomes were (self-)assessed through online questionnaires**		
"like this: Patient Health Questionnaire-9 (PHQ-9), Generalized Anxiety Disorder 7-item (GAD-7) "		
**4b-ii) Report how institutional affiliations are displayed**		
**5) CONSORT: Describe the interventions for each group with sufficient details to allow replication, including how and when they were actually administered**		
**5-i) Mention names, credential, affiliations of the developers, sponsors, and owners**		
**5-ii) Describe the history/development process**		
**5-iii) Revisions and updating**		
**5-iv) Quality assurance methods**		
**5-v) Ensure replicability by publishing the source code, and/or providing screenshots/screen-capture video, and/or providing flowcharts of the algorithms used**		
**5-vi) Digital preservation**		
**5-vii) Access**		
Not applicable, participants didn't need to pay for the AI chatbot and they had access through the WhatsAPP		
**5-viii) Mode of delivery, features/functionalities/components of the intervention and comparator, and the theoretical framework**		

"like this: AI Chatbot development procedures part"		
**5-ix) Describe use parameters**		
**5-x) Clarify the level of human involvement**		
**5-xi) Report any prompts/reminders used**		
"like this: Those who did not respond immediately would receive reminders until they completed the post-questionnaires. Participants with risky mental health problems were followed up by phone calls and were encouraged to ask for necessary medical services and mental health clinical departments. "		
**5-xii) Describe any co-interventions (incl. training/support)**		
Not applicable, no co-interventions were conducted		
**6a) CONSORT: Completely defined pre-specified primary and secondary outcome measures, including how and when they were assessed**		
"like this: Patient Health Questionnaire-9 (PHQ-9) is a self-assessment tool consisting of 9 questions that measure the frequency and severity of depressive symptoms over the past 2 weeks. Each question is derived from the Diagnostic and Statistical Manual of Mental Disorders-IV (DSM-IV) criteria and is rated on a scale of 0 (not at all) to 3 (almost daily) [13]. Generalized Anxiety Disorder 7-item (GAD-7) is a 7-item self-report scale that evaluates the frequency and severity of anxious thoughts and behaviors during the last 2 weeks. Items are based on the diagnostic criteria of the DSM-IV and scored from 0 (not at all) to 3 (nearly every day) [14]. Service Satisfaction Survey is a 3-item questionnaire for participants to report their degree of satisfaction after using the two platforms. The rating score ranges from 0 to 10, representing an increasing satisfaction from “Not at all likely” to “Extremely likely”. Based on the standardized cutoff that determines the severity of anxiety and depression, participants were divided into two groups: the no-risk group (total score below 4) and the risk group (total score above 4) "		
**6a-i) Online questionnaires: describe if they were validated for online use and apply CHERRIES items to describe how the questionnaires were designed/deployed**		
**6a-ii) Describe whether and how “use” (including intensity of use/dosage) was defined/measured/monitored**		
**6a-iii) Describe whether, how, and when qualitative feedback from participants was obtained**		
**6b) CONSORT: Any changes to trial outcomes after the trial commenced, with reasons**		
"like this: Participants were parents recruited through two school principal’s networks. Invitation letters were sent to kindergarten and primary school principal groups from chief schools, and 124 parents responded to this study and provided an electronic consent form."		
**7a) CONSORT: How sample size was determined**		
**7a-i) Describe whether and how expected attrition was taken into account when calculating the sample size**		
**7b) CONSORT: When applicable, explanation of any interim analyses and stopping guidelines**		
"like this: Patient Health Questionnaire-9 (PHQ-9) is a self-assessment tool consisting of 9 questions that measure the frequency and severity of depressive symptoms over the past 2 weeks. Each question is derived from the Diagnostic and Statistical Manual of Mental Disorders-IV (DSM-IV) criteria and is rated on a scale of 0 (not at all) to 3 (almost daily) [13]. Generalized Anxiety Disorder 7-item (GAD-7) is a 7-item self-report scale that evaluates the frequency and severity of anxious thoughts and behaviors during the last 2 weeks. Items are based on the diagnostic criteria of the DSM-IV and scored from 0 (not at all) to 3 (nearly every day) [14]. Service Satisfaction Survey is a 3-item questionnaire for participants to report their degree of satisfaction after using the two platforms. The rating score ranges from 0 to 10, representing an increasing satisfaction from “Not at all likely” to “Extremely likely”. Based on the standardized cutoff that determines the severity of anxiety and depression, participants were divided into two groups: the no-risk group (total score below 4) and the risk group (total score above 4) "		
**8a) CONSORT: Method used to generate the random allocation sequence**		
Random allocation sequence was generated by the RA with the computer		
**8b) CONSORT: Type of randomisation; details of any restriction (such as blocking and block size)**		
"like this: Participants were then randomly allocated with a 1:1 ratio into either the AI chatbot or the nurse hotline group with a block randomization of four."		
**9) CONSORT: Mechanism used to implement the random allocation sequence (such as sequentially numbered containers), describing any steps taken to conceal the sequence until interventions were assigned**		
Random allocation sequence was kept by the research staff who conducted the randomization.		
**10) CONSORT: Who generated the random allocation sequence, who enrolled participants, and who assigned participants to interventions**		
A research staff conducted the random allocation sequence, and another two research staffs enrolled participants and assigned participants. They were blinded to each other.		
**11a) CONSORT: Blinding - If done, who was blinded after assignment to interventions (for example, participants, care providers, those assessing outcomes) and how**		
**11a-i) Specify who was blinded, and who wasn’t**		
The study was not blinded to the participants, but it was blinded to research staff who conducted randomization and enrollment.		
**11a-ii) Discuss e.g., whether participants knew which intervention was the “intervention of interest” and which one was the “comparator”**		
**11b) CONSORT: If relevant, description of the similarity of interventions**		
Both can provide information about the Covid-19 vaccine to participants		
**12a) CONSORT: Statistical methods used to compare groups for primary and secondary outcomes**		
"like this: Smirnov test. Normal continuous variables were expressed as mean ± standard deviation (SD), and categorical variables were expressed as numbers and percentages. A per-protocol (PP) analysis was performed for all outcomes when comparing the two groups. The independent t-test was employed to compare the pre-anxiety/depression, post-anxiety/ depression, pre-post difference scores, and service satisfaction between the AI chatbot and Nurse hotline groups, respectively. The paired t-test was used to compare scores before and after group chat. Linear regression was conducted to analyze the difference in post-test scores between the AI chatbot and the nurse hotline adjusted by pre-test scores. Categorical variables were compared with the Chi-squared test. All the analyses were conducted using SPSS version 29.0."		
**12a-i) Imputation techniques to deal with attrition / missing values**		
Not applicable, and we used the participants' data who filled out the questionnaire both before and after the intervention		
**12b) CONSORT: Methods for additional analyses, such as subgroup analyses and adjusted analyses**		
No methods for additional analyses, such as subgroup analyses and adjusted analyses		
RESULTS		
**13a) CONSORT: For each group, the numbers of participants who were randomly assigned, received intended treatment, and were analysed for the primary outcome**		
"like this: Figure 1, Diagram of the study design."		
**13b) CONSORT: For each group, losses and exclusions after randomisation, together with reasons**		
"like this: Figure 1, Diagram of the study design."		
**13b-i) Attrition diagram**		
**14a) CONSORT: Dates defining the periods of recruitment and follow-up**		
"like this:From Oct 2022 to March 2023, all the participants (N=124) answered pre-questionnaires, and 62 participants in the AI chatbot group and 41 in the nurse hotline group completed both the pre-and post-questionnaires (Figure 1). Participants immediately filled out the post-test questionnaires after communicating with the AI chatbot or nurse. Those who did not respond immediately would receive reminders until they completed the post-questionnaires. "		
**14a-i) Indicate if critical “secular events” fell into the study period**		
**14b) CONSORT: Why the trial ended or was stopped (early)**		
Not applicable, the Covid-19 epidemic ended		
**15) CONSORT: A table showing baseline demographic and clinical characteristics for each group**		
"like this: Comparison of the anxiety and depression scores within groups before and after the test."		
**15-i) Report demographics associated with digital divide issues**		

Not applicable. No demographic data was collected for the privacy reasons		
**16a) CONSORT: For each group, number of participants (denominator) included in each analysis and whether the analysis was by original assigned groups**		
**16-i) Report multiple "denominators" and provide definitions**		
Analysis was conducted in participants completed the questionniare both before and after the intervention (62 in the AI chatbot group, 41 in the nurseline group).		
**16-ii) Primary analysis should be intent-to-treat**		
**17a) CONSORT: For each primary and secondary outcome, results for each group, and the estimated effect size and its precision (such as 95% confidence interval)**		
The independent t-test was employed to compare the pre-anxiety/depression, post-anxiety/ depression, pre-post difference scores, and service satisfaction between the AI chatbot and Nurse hotline groups, respectively. The paired t-test was used to compare scores before and after group chat. Linear regression was conducted to analyze the difference in post-test scores between the AI chatbot and the nurse hotline adjusted by pre-test scores.		
**17a-i) Presentation of process outcomes such as metrics of use and intensity of use**		
**17b) CONSORT: For binary outcomes, presentation of both absolute and relative effect sizes is recommended**		
Not applicable, no binary outcomes were collected.		
**18) CONSORT: Results of any other analyses performed, including subgroup analyses and adjusted analyses, distinguishing pre-specified from exploratory**		
Not applicable, no other analysis were performed		
**18-i) Subgroup analysis of comparing only users**		
**19) CONSORT: All important harms or unintended effects in each group**		
No important harms or unintended effects in each group		
**19-i) Include privacy breaches, technical problems**		
**19-ii) Include qualitative feedback from participants or observations from staff/researchers**		
DISCUSSION		
**20) CONSORT: Trial limitations, addressing sources of potential bias, imprecision, multiplicity of analyses**		
**20-i) Typical limitations in ehealth trials**		
"like this:Though AI chatbot shows its complement function in mental health and health-related fields, there are still some considerations about the drawbacks. For example, the safety and privacy concerns remain unclear [25]. Chatbots are unable to deliver the emotional support and personal bond that a certified mental health expert can give. They also cannot supply a diagnosis or management strategy for mental health disorders. Therefore, it is crucial to utilize AI chatbots as a complement to, rather than a substitute for, specialized mental health services. Moreover, more practice should be done to refine the AI chatbot to establish an integrated system for recognition-alert-reporting mental health problems. After early detection and identification of depression and anxiety, the AI chatbot should be designed to automatically send self-regulation advice to users to handle their negative emotions as well as send reminder messages to encourage them to seek medical services. Besides, the question coverage of the AI chatbot is too limited. The AI chatbot covers common mental health-related questions that may overlook the scope of other health problems. The breadth of the population should be enlarged in the public. Further studies are required to draw solid conclusions about the effectiveness and safety of chatbots. "		
**21) CONSORT: Generalisability (external validity, applicability) of the trial findings**		
**21-i) Generalizability to other populations**		
**21-ii) Discuss if there were elements in the RCT that would be different in a routine application setting**		
**22) CONSORT: Interpretation consistent with results, balancing benefits and harms, and considering other relevant evidence**		
**22-i) Restate study questions and summarize the answers suggested by the data, starting with primary outcomes and process outcomes (use)**		
"like this: This is among the first studies in Hong Kong that compare the effectiveness of AI chatbots and nursing hotlines in addressing mental health-related inquiries. The AI chatbot proved effective in reducing the level of anxiety and depression during the COVID-19 outbreak and is comparable with the conventional nursing hotline. The study suggests the potential use of AI chatbots as a complementary approach in mental health interventions when AI chatbots can provide more timely and non-interrupted 24-hour support to users in need during the challenging pandemic."		
**22-ii) Highlight unanswered new questions, suggest future research**		
Other information		
**23) CONSORT: Registration number and name of trial registry**		
Clinicaltrials.gov NCT06621134 (https://clinicaltrials.gov/study/NCT06621134)		
**24) CONSORT: Where the full trial protocol can be accessed, if available**		
No full trial protocol can be accessed.		
**25) CONSORT: Sources of funding and other support (such as supply of drugs), role of funders**		
"like this: This study was funded by the Collaborative Research Fund, University Grants Committee (Reference No.C7149-20GF). "		
**X26-i) Comment on ethics committee approval**		
**x26-ii) Outline informed consent procedures**		
**X26-iii) Safety and security procedures**		
**X27-i) State the relation of the study team towards the system being evaluated**		
